# Access: Is it all about costs?

**DOI:** 10.1016/j.omton.2026.201372

**Published:** 2026-11-06

**Authors:** Marcos de Lima, David Wald

**Affiliations:** 1 The Ohio State University, Columbus, OH, USA; 2 Case Western Reserve University School of Medicine, Cleveland, OH, USA

## Main text

As reported in the previous issue of *Molecular Therapy Oncology*, Suppipat in Thailand used the automated closed system (CliniMACS Prodigy, Miltenyi Biotec) to manufacture anti-CD19 chimeric antigen receptor (CAR T) cells to successfully treat 12 patients with diffuse large B cell lymphoma (DLCL).^1^ The authors report one-year event-free and overall survival of 58.3% and 90%, respectively, at a median total cost of USD 111,231. Patients’ treatment was financially supported by government grants and philanthropic contributions.

The outcomes were similar to what would be expected after treatment with commercially available CAR T products. It again illustrates the curative potential of this technology for a significant fraction of otherwise incurable lymphomas. Although with any new technology there are needed improvements, the main issue that has risen after a decade or so of CAR T cell development is the profound inequality of access to it. The available data is sobering. Take two recently published reports (out of a rapidly growing body of evidence).

Chung et. al looked at patients older than 65 years with DLCL in third line of treatment included in the United States Surveillance, Epidemiology, and End Results-Medicare Data (*n* = 2,241).^2^ Only 5.4% of eligible patients received CAR T cells. Utilization inversely correlated with greater geographic distance to treatment centers and higher uninsured or Medicaid-dominant rates. In addition, lower county median household income correlated with lower treatment rates.

Living in high income countries does not guarantee access, as further illustrated by Cliff and colleagues who reported on a cross-sectional analysis of G20 countries health technology assessment decisions.^3^ Less than half of 18 U.S. Food and Drug Administration-approved CAR T indications are publicly reimbursed outside the USA. Fewer CAR T products are reimbursed in Australia and Canada, for example, than in the US, France, and Germany, and even fewer in Singapore, South Korea, and Brazil. In the latter, fewer than 200 patients with lymphoma have been treated (Fernando Barroso, MD, personal communication). Among the countries surveyed, 48% of CAR-T product-indication pairs were accepted for reimbursement. Approval was followed by a median time of 1.54 years to actual access. Interestingly, recommendation by the respective health authorities was not the main barrier. Road blocks included manufacturers’ decision to not seek reimbursement in certain markets, and questions regarding cost-effectiveness and overall costs of care that further limited incorporation of these treatments. In the US, an analysis of 863 insurance claims indicated that the average allowed cost for a 60-day episode (including the product and associated clinical care) is above USD 800,000.^4^ Needless to say, price matters.^5^

It is undeniable that the cellular biologic and therapeutic revolution we are witnessing has been enabled by big pharma. Centralized manufacturing has allowed thousands of products to be produced reproducibly and safely. On the other hand, this model is associated with the disparities discussed earlier. Suppipat et. al contribute to this conversation by demonstrating a potential contribution to increase access. It comes in different names, such as point (or place) of care or academic CAR T. The results reported here stem from an industry-institutional collaboration, a model that we and others have investigated in the last 8 years.^6^ In our experience, using the same lentiviral vector used by Suppipat and colleagues and the same platform in two subsequent studies, we saw consistency in release criteria and other comparability parameters between our laboratory in Cleveland, US and in Moscow, Russia, and subsequently between Cleveland and Saint Louis, US. These results were an initial message that we hope is reaching regulatory agencies—that it is possible to decentralize manufacturing while retaining adequate expected safeguards. The Barcelona group has now shown the feasibility of a regional approach to decentralized CAR T production, complete with regulatory approval for limited commercialization assuring sustainability.^7^ Here, the European model of hospital exception is quietly enabling expansion of point-of-care manufacturing. Regretfully, this model is yet to be adopted in the US, for example. The main highlight is the possibility of charging for the locally made CAR T cells, therefore facilitating and nurturing such projects.

Brazil is also seeking local solutions. The group at Ribeirao Preto is conducting a multi-center study of a CD19 CAR T seeking registration and inclusion in the country public health care system, where it will be offered free of cost (with centralized manufacture). Caring Cross (US) is transferring technology to Biomanguinhos (Rio de Janeiro) that will allow Biomanguinhos to produce vectors and cells at the point of care. The first vector is under investigation by our group at Ohio State University in a phase 1 study that will subsequently be transferred to Brazil.^8^^,^^9^ Elsewhere, CAR T cells developed in India and China are also contributing to this expansion, although not all projects involve place-of-care production.

Governments of course have a key role. They may finance development and care delivery, but they may also inadvertently delay or block local innovation by excessive regulatory burden imposed over a fragile innovation ecosystem. High taxes on, and obstacles to importing reagents is another common barrier.

Suppipat and colleagues showed feasibility, reproducibility, and safety in this study. The next question for them, and for our field, is how to assure that this momentum is not lost and that it does not stay limited to a small clinical trial? One major obstacle that still needs to be addressed is how to effectively scale cost-effective manufacturing so that more than a tiny fraction of eligible patients can benefit. There is no doubt that it is not all about cost and pricing, but access to modern therapies such as CAR T cells is not going to increase solely based on traditional centralized manufacturing. It is possible that *in vivo* CAR T approaches, allogeneic platforms, and faster and fully automated *ex vivo* manufacturing will reduce production costs.^10^ But if followed by similar pricing structures, we will remain where we are now. At the end, it is not necessarily one model versus other (centralized vs. point of care). It is a conversation about multiple models of delivery that must complement each other.

## Declaration of interests

The authors have no competing interests to declare.
